# Harnessing yeast biodiversity for advanced industrial applications

**DOI:** 10.1093/femsyr/foag031

**Published:** 2026-08-22

**Authors:** Cheng Cheng, Yu-Zhen Li, Zhenzhi Wang, Hui Chen, Bei Liao, Kai Li, Xin-Qing Zhao

**Affiliations:** School of Biotechnology and Food Engineering, Hefei Normal University, Hefei 230601, China; State Key Laboratory of Microbial Metabolism, School of Life Sciences and Biotechnology, Shanghai Jiao Tong University, Shanghai 200240, China; State Key Laboratory of Microbial Metabolism, School of Life Sciences and Biotechnology, Shanghai Jiao Tong University, Shanghai 200240, China; Hubei Provincial Key Laboratory of Yeast Function, Angel Yeast Co., Ltd., Yichang, Hubei Province 443002, China; Hubei Provincial Key Laboratory of Yeast Function, Angel Yeast Co., Ltd., Yichang, Hubei Province 443002, China; State Key Laboratory of Microbial Metabolism, School of Life Sciences and Biotechnology, Shanghai Jiao Tong University, Shanghai 200240, China; State Key Laboratory of Microbial Metabolism, School of Life Sciences and Biotechnology, Shanghai Jiao Tong University, Shanghai 200240, China

**Keywords:** yeast, biodiversity, metabolic engineering and synthetic biology, artificial intelligence, industrial applications

## Abstract

Budding yeast *Saccharomyces cerevisiae* and various non-conventional species are widely applied in the production of diverse industrial products, including pharmaceuticals, biofuels, biochemicals, and biomaterials, due to their rich natural and engineered biodiversity. This review summarizes recent advances in harnessing yeast biodiversity for industrial applications, including natural yeast diversity derived from ecological adaptation and genetic variation across diverse environments, and artificial diversity generated through adaptive laboratory evolution, metabolic or epigenetic engineering, and synthetic biology. We highlight the establishment of strain-level culture libraries and extremophilic yeasts as models for stress adaptation. In addition, we emphasize that the integration of multi-omics analysis, AI-assisted enzyme and strain engineering, and process optimization strategies are critical for the fully exploration and utilization of yeast biodiversity for efficient bioproduction.

## Teaser

This review integrates natural and engineered yeast biodiversity with future strain repositories, AI driven design, and process optimization for sustainable biomanufacturing.

## Introduction

Yeasts are unicellular fungi that reproduce through fission or budding (Rose and Harrison 1987), and are broadly categorized into conventional yeasts including the genera *Saccharomyces* and *Schizosaccharomyces*, and non-conventional yeasts which encompass all other yeast species. Yeasts have been isolated not only in fermentation environments but also across a wide range of natural habitats, demonstrating notable ecological diversity. Since 1987, ecological niches of yeasts have been systematically explored, revealing their remarkable environmental adaptability (Phaff and Starmer 1987). Advances in molecular identification and whole–genome sequencing have greatly expanded our understanding of yeast biodiversity. By 2011, more than 1500 yeast species had been identified (Kurtzman et al. 2011). In addition to the model organism *Saccharomyces cerevisiae*, numerous non-conventional yeasts such as *Rhodosporidium toruloides, Komagataella phaffii* (formerly *Pichia pastoris*), *Kluyveromyces marxianus*, and *Yarrowia lipolytica* have been isolated and characterized from various ecosystems.

Non-conventional yeasts often exhibit excellent industrial traits, including high stress tolerance, unique substrate metabolism, and superior production capability (Bai et al. 2023). Moreover, culturable natural yeast strains serve as foundational resources for genetic modification, providing valuable regulatory elements, functional components, and novel enzyme-encoding genes. However, the productivity of most natural yeast strains is constrained by inefficient substrate conversion and significant byproduct accumulation. To satisfy industrial requirements, the diversity of yeast is expanded not only by exploring natural capacity but also by integrating non-rational and rational strategies through artificial engineering. Non-rational approaches, such as adaptive laboratory evolution (ALE) and random mutagenesis, generate strains with improved traits through iterative stress selection without prior genetic knowledge (Pereira et al. 2020, Fernandes et al. 2023). Rational approaches include targeted metabolic engineering, CRISPR-based genome editing, and pathway optimization for precise genetic modulation (Lian et al. 2018, Zhang et al. 2021). Notably, multi-omics analyses and artificial intelligence are increasingly integrated to accelerate strain engineering (Itto-Nakama et al. 2021, Kotopka and Smolke 2020, My et al. 2025), and the synergy between these strategies has significantly advanced the construction of robust yeast cell factories.

Both conventional and non-conventional yeast exhibit great potential in industrial production. For instance, *S. cerevisiae* has been engineered to synthesize pharmaceuticals and high-value natural products (Li et al. 2025a, Liu et al. 2025a, Qiu et al. 2025). Some non-conventional yeasts possess broad substrate utilization capabilities, unique physiological traits, and stress tolerance, making them well-suited to produce antibodies, terpenoids, and biomaterials, etc. (Gómez-Ramírez et al. 2023, Tian et al. 2024, Tong et al. 2025). However, so far only limited yeast species have been applied in industrial application. Major challenges include the difficulty of genetic modification in non–conventional yeasts, narrow carbon utilization spectra, limited knowledge of metabolic regulation, and a lack of large–scale fermentation experience. This review summarizes the species and genetic diversity of naturally isolated yeasts, highlights recent progress in mining natural strains and engineering artificial diversity, and discusses emerging strategies in metabolic engineering and laboratory evolution. By elucidating the ecological and genetic bases of yeast diversity, we aim to facilitate the rational design and construction of microbial cell factories for sustainable industrial applications.

## Natural diversity of yeast

### Species diversity

Yeasts exhibit remarkable species diversity across natural environments. Advances in classification and identification technologies have prompted the discovery of an increasing number of yeast species, providing fundamental support for strain chassis development and industrial applications.

Genomic analysis revealed that the global genetic diversity of *S. cerevisiae* primarily originates from Far Eastern Asian strains, with ancient basal lineages exclusively identified in China (Bai et al. 2022). Over the past two decades, 107 novel Agaricomycotina species and 26 Ascomycota genera have been isolated through systematic environmental surveys (Li et al. 2020), while 392 yeast strains spanning 20 genera were identified through investigations of intertidal ecosystems, with 17 species first discovered in marine environments (Xue et al. 2024). In our previous studies, non-conventional yeasts from Tibetan lichens, including lipid-producing *Curvibasidium* sp. and ergothioneine-synthesizing *Rhodotorula* sp., demonstrated significant application potential (Bai et al. 2023, Li et al. 2025b).

Global exploration continues to broaden our understanding of yeast species diversity. Novel basidiomycetes have been isolated from Italian fruit substrates and Mexican desert vineyards (Lorenzini et al. 2019, Perrusquía-Luévano et al. 2019). Brazilian rainforest soils and rotten wood harbor abundant yeast resources (Santos et al. 2023), with 569 strains isolated from the Amazon, including 20 novel xylose-fermenting species (Barros et al. 2023). Additionally, multiple cellobiose-fermenting yeasts producing β-glucosidase and ethanol have been obtained from Brazilian ecosystems (Lopes et al. 2018). Yeast strains separated from coconut and sugarcane residues exhibit naturally mixed-sugar metabolism or lipid and biodiesel production capabilities (Cheng et al. 2018, Watsuntorn et al. 2021). The continuous discovery of yeast strains with valuable physiological and metabolic traits further enhances their potential for diverse biotechnological and industrial applications.

### Ecological adaptability

Yeast species isolated from distinct ecological niches exhibit remarkable biodiversity. Previous studies have revealed diverse yeast communities in traditional fermented beverages, on wine grape surfaces or plant leaves (Chai et al. 2024, Ferluga et al. 2024, Shibayama et al. 2024), demonstrating the ability of yeasts to adapt to diverse environments, even in extreme conditions such as thermal stress, salt stress, and acidic environments.

Chinese researchers isolated 194 yeast strains from the Qaidam Basin, which is one of Earth’s highest, largest, and driest deserts, and considered as a Mars analog area. Total of 21 species across 12 genera were identified, including 19 basidiomycetous yeasts and 5 novel species such as *Filobasidium chaidanensis* and *Kondoa globosum*. Notably, these soil-derived yeast strains exhibited stronger salt tolerance than those from plants or lichens (Wei et al. 2022). Extremophilic adaptations are also observed in hypersaline conditions (Andreu et al. 2022), as demonstrated by halophilic yeasts characterized in Botswana’s Makgadikgadi and Sua pans (Loeto et al. 2021). Black yeasts, known for stress tolerance and survival in extreme habitats (Coleine et al. 2022), also exhibit distinctive production capabilities, as demonstrated by a marine-derived strain that produces extracellular polysaccharides (Zaghloul et al. 2024). Cold-adapted yeasts have been isolated from high-altitude volcanic soils in Mexico (Tapia-Vázquez et al. 2020), while symbiotic associations between lichens and basidiomycetous yeasts occur in complex mountainous terrains (Cometto et al. 2022).

Both industrial and geological extreme environments serve as valuable sources of microbial resources. A strain from sparkling wine production exhibited extraordinary acetic acid resistance (Mira et al. 2014), while acidophilic isolates were separated from an ultra-acidic, heavy metal-contaminated mine in Portugal (Gadanho and Sampaio 2009). Interestingly, yeasts have been isolated from organisms such as beetle guts (Yoshihashi and Degawa 2023), and a natural yeast, *Trichosporon asahii* HZ10 from honeycomb, exhibits robust flavonoid production ability (Xue et al. 2023). Collectively, extremophilic yeasts are emerging as next–generation eukaryotic models for understanding systems–level stress adaptation (Padilla-Garfias and Peña 2026).

### Genetic and phenotypic diversity

Wild yeast strains exhibit unique genetic characteristics (Hittinger et al. 2015). For instance, *S. cerevisiae, S. uvarum*, and *S. kudriavzevii* display distinct nitrogen metabolism patterns (Minebois et al. 2025), while peroxisomal membrane proteins differ significantly between *S. cerevisiae* and *S. paradoxus* (Dubin et al. 2020). These species-specific traits contribute to ecological adaptation and industrial performance.

In the aspect of lignocellulose-derived bioethanol and value-added chemical production, genomic characterization of 20 *S. cerevisiae* strains identified copy number variations (CNVs) and single-nucleotide polymorphisms (SNPs) correlating with distinct acetic and formic acid tolerance (My et al. 2025). Xylose constitutes roughly 35% of the total sugar content in lignocellulosic biomass, and *S. cerevisiae* was traditionally considered incapable of native xylose metabolism. However, a naturally xylose-utilizing strain, YB-2625, isolated from sugarcane bagasse, efficiently co-consumes mixed sugars (Cheng et al. 2018), while strain 202–3 from Colombia metabolizes xylose to xylitol and exhibits high inhibitor tolerance (Lagos et al. 2023). Besides *S. cerevisiae*, non-conventional yeasts like *Candida sojae* and *Spathaspora marinasilvae* demonstrate xylose-to-xylitol and ethanol conversion capabilities (Kusumawati et al. 2023, Barros et al. 2024). In addition, oleaginous yeasts including *Rhodotorula toruloides* and *Aureobasidium melanogenum* could produce lipids and mannitol-rich polyol esters from diverse carbon sources (Saika et al. 2020, Qvirist et al. 2022).

The genomes of natural isolates also serve as reservoirs of functional genetic elements and enzyme-coding genes. In *R. toruloides*, constitutive promoters with over 100-fold strength variation were identified (Guo et al. 2023), and multiple strong endogenous promoters were characterized in *K. phaffii* (Dou et al. 2021). Additionally, a robust signal peptide from *Y. lipolytica* enhances protein secretion efficiency (Celińska et al. 2018), whereas the OST1-derived signal peptide from *S. cerevisiae* facilitates lytic polysaccharide monooxygenase (LPMO) secretion, which is critical for biomass conversion (Rieder et al. 2021). Yeast genomes are valuable reservoirs for industrial enzymes. Genome mining of 250 fungal genomes identified 150 putative genes, 18 of which exhibited glucuronoyl esterase activity for biomass saccharification (Dilokpimol et al. 2018). This genomic screening also uncovered multiple enzymes, including aldo-keto reductases from *Candida glabrata*, nine polyketide synthases from *Preussia isomera*, various sterol esterases and lipases (Barriuso et al. 2013, Basak et al. 2018, Liu et al. 2022), and endoinulinase from *Lipomyces starkeyi* for fructooligosaccharide synthesis (Bao et al. 2019). Systematic mining of such genetic elements facilitates metabolic engineering and strain optimization.

## Rational and non–rational approaches to expand yeast biodiversity

### Metabolic engineering and genome editing

**Figure 1:**
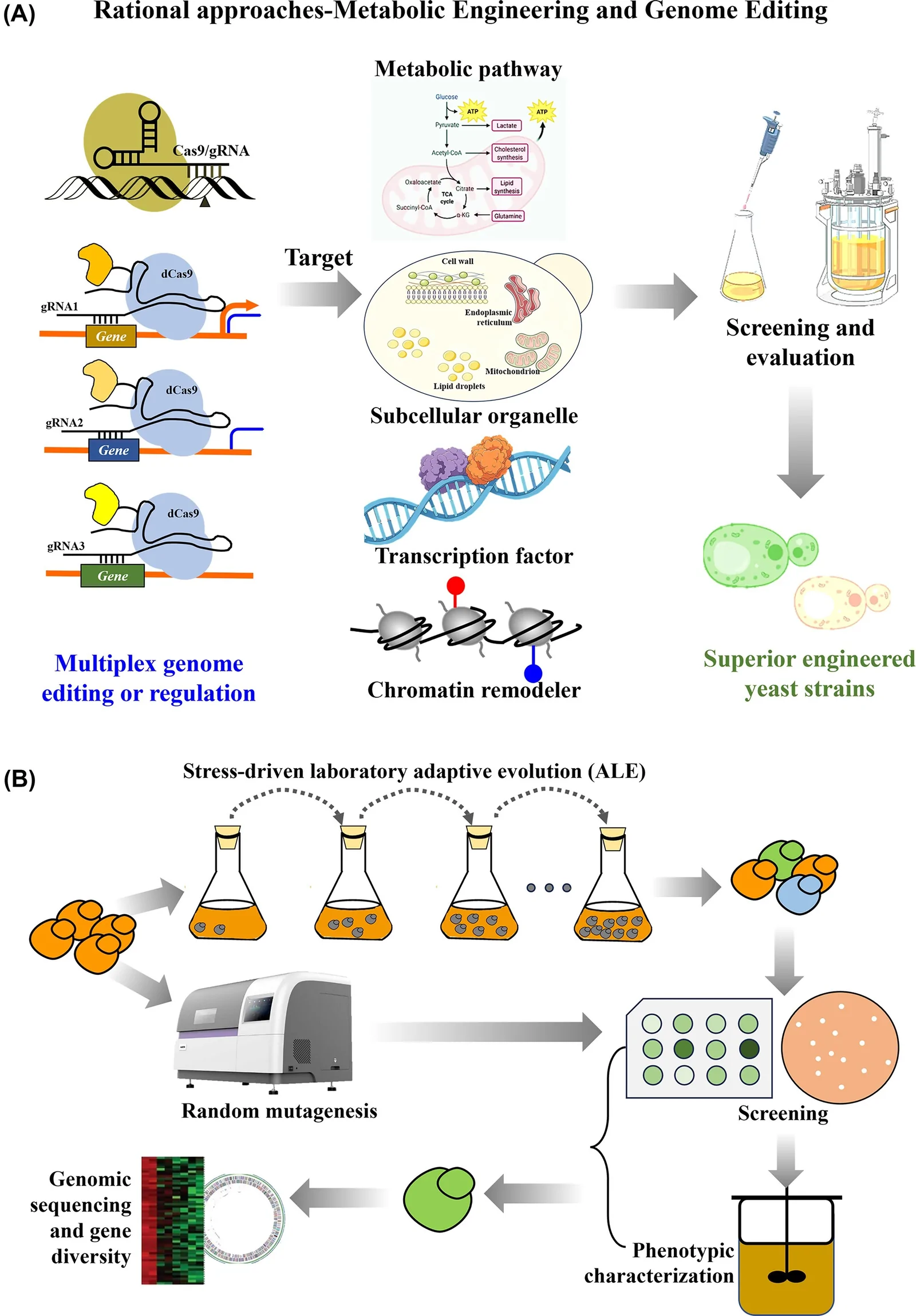
Rational and non–rational approaches for expanding yeast biodiversity and constructing superior cell factories. (A) Rational strategies such as metabolic engineering and genome editing precisely rewire cellular metabolism at the gene, pathway, chromosome, and organelle levels. (B) Non–rational approaches, including adaptive laboratory evolution (ALE) and random mutagenesis, generate genetic diversity through environmental stress or induced mutations, followed by selection to enrich strains with improved tolerance or productivity.

Metabolic engineering is a rational and effective strategy to develop cell factories for producing various products from renewable carbon sources (Fig. 1A). In *S. cerevisiae*, multiple strategies including promoter, terminator transcription factor and enzyme engineering have developed for strain modification (Lian et al. 2018). To accelerate strain cell factory optimization, multiplex genome editing has been applied to overcome bottlenecks in biosynthetic pathways (Zhang et al. 2021). Emerging strategies such as chromosome engineering and subcellular organelle engineering are gradually applied to the yeast modification. Chromosome engineering, especially when combined with CRISPR/Cas9 and SCRaMbLE system, enable rapid generation of structural variations (Luo et al. 2018, Jin et al. 2020). Subcellular organelle engineering, which synergistically modifies multiple organelles including mitochondria, the endoplasmic reticulum, lipid droplets, and the cell wall, has led to increased squalene production (Tang et al. 2025).

Metabolic engineering is also advancing in non-conventional yeasts including *Y. lipolytica, K. phaffii*, and *R. toruloides* (Ganesan et al. 2019, Zha et al. 2023), which partially solves the problem of difficult metabolic engineering in these hosts. For instance, CRISPR-assisted Cre/loxP system enabled high ergothioneine production in *R. toruloides* (Liu et al. 2024). Through these efforts, yeasts have been endowed with non-native carbon utilization and heterologous bioactive compound production (Ma et al. 2019, Srinivasan and Smolke 2020), expanding their physiological and metabolic diversity.

In addition to broadening the metabolic potential of yeast, metabolic engineering has been used to improve stress tolerance during industrial fermentation. Acetic acid is a primary inhibitor in lignocellulosic hydrolysates to hamper yeast growth and fermentation performance. Our studies identified several novel genes enhancing acetic acid tolerance, including *SET5, RTT109, HOG1*, and *INO80* (Cheng et al. 2016, Ye et al. 2022, Yuan et al. 2024, Zhang et al. 2024). Epigenetic regulation encompassing histone modification and chromatin remodeling, has emerged as a promising strategy for improving yeast robustness and metabolic ability. Disruption of the histone H3 acetyltransferase Rtt109 increased stress response gene expression and antioxidant enzyme activities (Cheng et al. 2016). Overexpression of the chromatin remodeler Ino80 improved acetic acid tolerance and coordinated carbon and nitrogen metabolism (Yuan et al. 2024). Similarly, deletion of *NGG1*, which encodes a component of the SAGA transcriptional coactivator complex, enhanced xylose utilization and remodeled amino acid metabolism, highlighting the role of chromatin modification in balancing carbon and nitrogen fluxes (Cheng et al. 2022). Recently, a systematic TF phenotyping study in *Y. lipolytica* revealed stress-specific modulation patterns, in which tailored manipulation of individual TFs conferred differential benefits against heat, osmotic stress, lactic acid, and 2-phenylethanol (Gorczyca et al. 2026). Collectively, these findings demonstrate that targeted genetic manipulation, including epigenetic and transcription factor engineering, provides effective strategies for enhancing yeast robustness in biorefining processes.

### Adaptive laboratory evolution

Random mutagenesis and adaptive laboratory evolution (ALE) have been widely used as non-rational strategy for expanding yeast diversity (Arora et al. 2020). Random mutagenesis does not require extensive genomic pre–knowledge and offers a simple, cost–effective route to generate beneficial genetic diversity for strain development, whereas ALE utilizes non-rational, selection-driven evolution to enhance stress tolerance and improve other industrially relevant traits (Johnson et al. 2021, Wan et al. 2024) (Fig. 1B). ALE is broadly applicable to both conventional and non-conventional yeasts (Fernandes et al. 2023), enabling rapid development of high-performance strains for biofuel and biochemicals production. Moreover, when integrated with omics, ALE facilitates revealing molecular mechanisms and identify engineering targets (Mans et al. 2018). For instance, ALE-derived *S. cerevisiae* mutants tolerant to coumaric and ferulic acids harbored point mutations in the transcriptional activator Aro80, revealing that the Aro80-regulated gene *ESBP6* is key to aromatic acid efflux and tolerance (Pereira et al. 2020). In another study, combining ALE with omics revealed genetic determinants of glycerol catabolism and adaptive trade-offs in *S. cerevisiae* (Strucko et al. 2018).

ALE also serves as a platform for improving tolerance to other lignocellulosic hydrolysate inhibitors (Menegon et al. 2022). Enhanced vanillic acid tolerance in *Y. lipolytica* was achieved via mutations in RNA processing and multidrug transporter pathways (Sha et al. 2023). Similarly, *R. toruloides* evolved over 16 rounds showed 2.5-fold higher furfural tolerance, enabling lipid production from agricultural waste (Park et al. 2023). ALE has also induced cell wall modifications in succinic acid-resistant *S. cerevisiae* (Qin et al. 2025a) and revealed phosphorylation and arginine rewiring in β-phenylethanol-tolerant strains (Yang et al. 2025a).

Furthermore, combining ALE with metabolic engineering has accelerated industrial strain development. High-yielding *S. cerevisiae* for L-lactic acid and ethyl hexanoate were obtained using this strategy (Guo et al. 2025, Yang et al. 2025b). Leveraging antioxidant properties, short-term H₂O₂-induced ALE enhanced carotenoid biosynthesis (Reyes et al. 2014). By combination of mutagenesis with H₂O₂-induced ALE to optimize the upstream metabolic flux toward farnesyl pyrophosphate (FPP), the highest lycopene titer was achieved in *S. cerevisiae* (Zhou et al. 2023). Additionally, ALE has been coupled with biosensor-based selection to enhance aromatic amino acid production in yeast, as demonstrated by growth-coupled enrichment of strains with improved tryptophan-derived metabolite titers (Sun et al. 2026).

In summary, both random mutagenesis and ALE contribute to constructing robust microbial cell factories to expand substrate utilization, enhance stress tolerance, and improve product titers (Jia et al. 2023). Notably, suitable environmental or nutritional stress strategies should be adopted to rapidly obtain the dominant strain. Moreover, the long-term genetic and phenotypic stability of evolved strains remains a key issue that requires further investigation.

## Application of yeast diversity

### Multi-substrate fermentation

**Figure 2:**
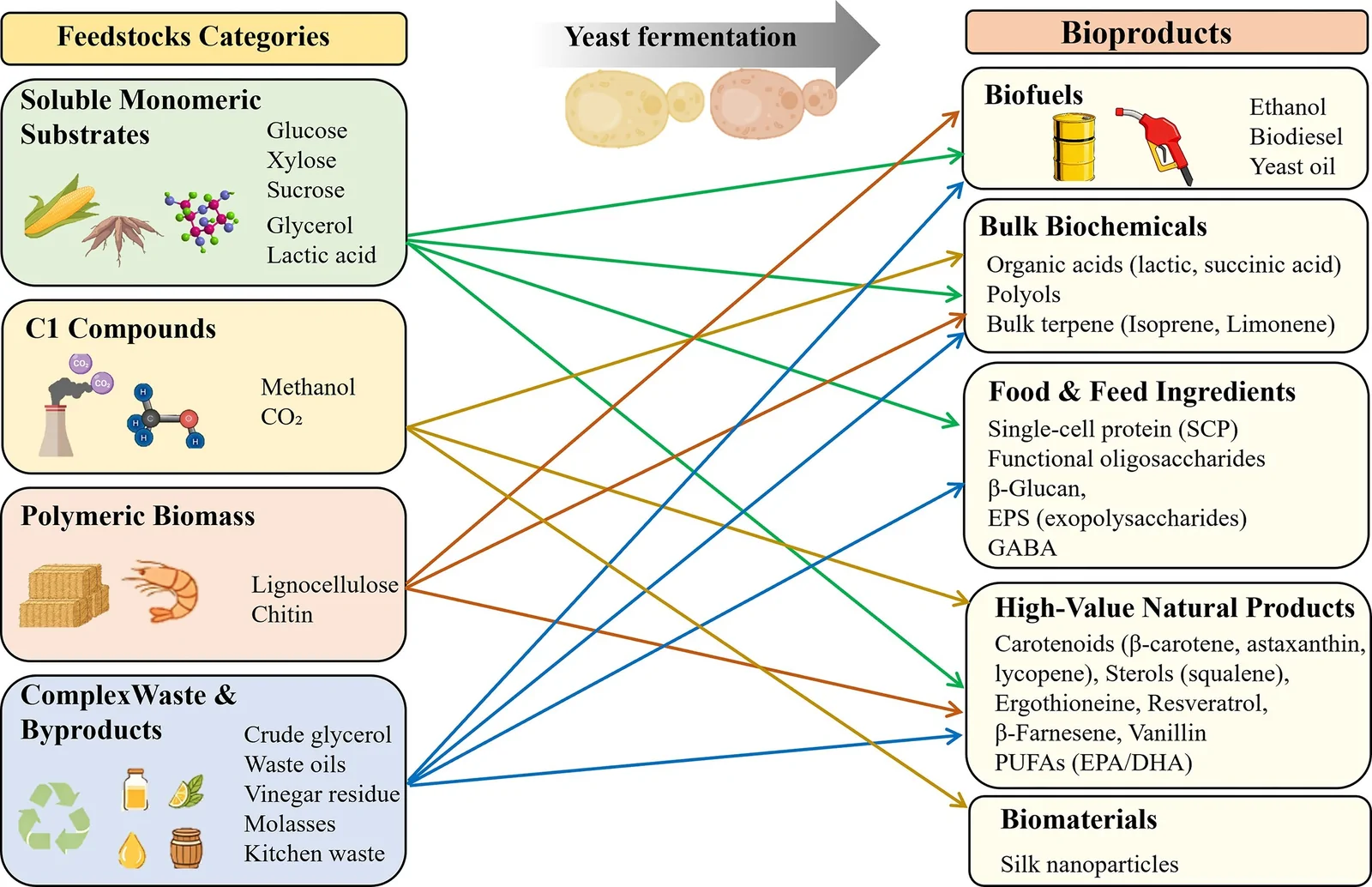
Conversion of diverse feedstocks to value–added products by yeast strains. Yeast strains utilize a broad range of renewable and waste-derived feedstocks, including sugars, glycerol, acetate, C1 compounds, and lignocellulosic or food industry residues. These substrates are converted into various products, such as biofuels, biochemicals, single-cell protein, and high-value natural compounds.

Yeasts are vital industrial microorganisms capable of utilizing diverse substrates (Fig. 2). This substrate diversity enhances ecological adaptability and industrial potential. For example, the oleaginous yeast *R. toruloides* could utilize glucose, xylose or acetate as the sole carbon source to synthesize fatty acids (Reķēna et al. 2025). Engineered *K. marxianus* utilized glucose or glycerol as substrates for inositol production (Lan et al. 2025).

More low-cost and waste substrates are increasingly exploited for yeast fermentation. Both *S. cerevisiae* and *K. phaffii* could produce chitin and β-glucan with kitchen waste (Günal-Köroğlu et al. 2025). According to recent studies, purified biodiesel-derived crude glycerol has been used by *R. toruloides* to produce yeast oil and β-carotene (Khuntong et al. 2025). Waste oils such as cooking and motor oil are converted by *Y. lipolytica* into eicosapentaenoic acid and biodiesel (Miranda et al. 2024, Qin et al. 2025b). In addition, food industry residues like vinegar residue and sugarcane molasses were used to produce single-cell protein and exopolysaccharides (EPSs) (González-Torres et al. 2024, Li et al. 2025c). More studies focused on rich one-carbon feedstock utilization such as carbon dioxide and methanol, which is helpful to mitigate the climate change and realize carbon neutrality (Espinosa et al. 2020). *Ogataea polymorpha, Y. lipolytica*, and *K. phaffii* have been engineered to achieve effective utilization of methanol to generate β-farnesene, resveratrol and L-lactic acid, respectively (Li et al. 2024, Jiang et al. 2025, Wu et al. 2025).

In addition to these waste-derived and C1 feedstocks, lignocellulosic biomass represents another key renewable resource. *Saccharomyces cerevisiae* and *K. phaffii* are commonly employed to ferment lignocellulosic hydrolysate into bioethanol, L-lactic acid, or vanillin (Choi et al. 2024, Xin et al. 2025). Non-conventional yeasts like *Y. lipolytica* and *R. toruloides* show advantages in converting lignocellulose to value-added biofuels and chemicals (Koh et al. 2024). Chitin, the second most abundant biomass after cellulose, is composed of N-acetylglucosamine (NAG) units. The novel oleaginous yeast strain *Sakaguchia* sp. HKC2 accumulates Polyunsaturated Fatty Acids (PUFAs) and carotenoids using NAG as the carbon source, highlighting bioconversion potential of this aquatic by-products by yeasts (Pham et al. 2025).

Overall, with the continuous isolation of natural yeasts with novel performance and the construction of artificial cell factories, it provides more strategies to produce biofuels and biochemicals from industrial byproducts, wasted materials and sustainable resources.

### Yeast cell factories for high-value natural products

In addition to traditional applications in fermented foods and alcoholic beverages, yeast strains have emerged as powerful microbial chassis for synthesizing various high-value natural products through synthetic biology approaches, overcoming limitations of plant or animal systems such as low yields and complex pathways.

*S. cerevisiae* has been widely used for pharmaceutical production. Biosynthesis of progesterone has been achieved using engineered *S. cerevisiae* through heterologous expression of plant-derived pathways (Li et al. 2025a). A high-efficiency hemoglobin expression yeast platform was developed to achieve the production of functional human, porcine, and bovine hemoglobins, providing novel strategies for heteromultimeric protein synthesis (Liu et al. 2025a). Moreover, *S. cerevisiae* has been employed as a cell factory for serotonin production by integrating heterologous pathway expression with medium optimization, which laid the foundation for sustainable and eco-friendly industrial serotonin biosynthesis (Planells-Cárcel et al. 2025). Bicyclic monoterpenes like camphor and bornyl acetate were produced for the first time in *S. cerevisiae* (Tominaga et al. 2025). Recent advances have also enabled yeast to produce a range of tryptophan-derived compounds, including neuromodulator melatonin, hallucinogenic alkaloid psilocybin, and pigment violacein, highlighting the versatility of yeast as a platform for indole-based natural products (Sun et al. 2026). Additionally, yeast platforms enabled efficient production of oligosaccharides, triterpenoids, ergothioneine, and cordycepin (Chen et al. 2025, Liu et al. 2025b, Qiu et al. 2025).

Recent advances in metabolic engineering and synthetic biology have prompted the utilization of non-conventional yeast strains such as *R. toruloides, K. phaffii, K. marxianus, Y. lipolytica* in biofuel and biochemical production. Oleaginous yeast strains including *Y. lipolytica, Rhodotorula* sp., *R. toruloides* etc, are prominent for lipid and terpenoid production. *Yarrowia lipolytica*, a biosafe yeast, not only exhibits naturally excellent lipid accumulation capabilities (Lazar et al. 2018), but can also be modified to utilize various substrates to produce squalene (Chai et al. 2025) and antioxidant compounds such as lycopene, β-carotene, and astaxanthin (Mai et al. 2025). *Rhodotorula* sp. can accumulate over 70% lipids and utilize diverse carbon sources including glucose, xylose, volatile fatty acids, and industrial byproducts (Tong et al. 2025). What’s more, *Rhodotorula sp*. as well as *R. toruloides* is commonly employed for synthesis of terpenoid-based bioproducts (Adamczyk et al. 2025, Tong et al. 2025), which have broad application in food, pharmaceutical and cosmetic industries. Other oleaginous yeasts like *Cutaneotrichosporon oleaginosus* and *O. polymorpha* have been developed for fatty acid production, with engineered *O. polymorpha* co-utilizing glucose and xylose from lignocellulose hydrolysates (Ni et al. 2024, Duman-Özdamar et al. 2025).

*Kluyveromyces marxianus* is thermotolerant, fast-growing, and assimilates multiple substrates, enabling production of enzymes, flavor compounds, bioethanol, and lactic acid (Fonseca et al. 2008, Baptista and Domingues 2022, Cui et al. 2026). Recent advances highlight *K. marxianus* as a versatile food-grade host not only for recombinant protein expression but also for single-cell protein (SCP) production (Cui et al. 2026). With gene editing, it now produces inositol from starch (Lan et al. 2025) and carotenoids from corn cob hydrolysates (Yang et al. 2022). Genes involved in GABA metabolism were also identified in *K. marxianus* (Perpetuini et al. 2020). Meanwhile, *K. phaffii* is suitable for C1 feedstocks and high-value compound synthesis. Engineered strains produce malic acid and erythritol from methanol (Guo et al. 2021, Wang et al. 2025a), as well as pharmaceuticals including antibody fragments, monoterpene indole alkaloids, nutraceuticals such as myo-inositol and silk fibroin nanoparticles (Zhang et al. 2022, Tian et al. 2024). The key applications and features of representative yeast species are summarized in Table 1.

**Table 1:** Representative industrial applications and key features of selected yeast species discussed in this review.

Species	Applications and representative products	Notable features	References
*Saccharomyces cerevisiae*	Bioethanol, fermented beverages; pharmaceuticals (progesterone, hemoglobins, serotonin); high–value natural products (terpenoids, oligosaccharides, triterpenoids, ergothioneine, cordycepin)	Well-established genetic toolbox; biosafe; high-yield platform for diverse products	Li et al. 2025a, Liu et al. 2025a, Planells-Cárcel et al. 2025, Tominaga et al. 2025; Liu et al. 2025b, Qiu et al. 2025, Chen et al. 2025
*Yarrowia lipolytica*	Lipids, squalene, carotenoids (lycopene, β-carotene, astaxanthin); recombinant proteins	Naturally oleaginous; efficient protein secretion; engineered for diverse substrates and high-value products	Beopoulos et al. 2009, Ye et al. 2012, Lazar et al. 2018, Madzak 2018, Chai et al. 2025, Mai et al. 2025
*Rhodotorula* sp.	Lipids, terpenoids (carotenoids, β-carotene, astaxanthin, limonene, bisabolene); resveratrol; ergothioneine	High lipid yield; utilizes diverse low-cost carbon sources; environmental adaptability	Tong et al. 2025, Li et al. 2025b
*Rhodotorula toruloides*	Bisabolene; lipids; β-carotene	Broad substrate range; inhibitor tolerance; grows on various carbon sources	Qvirist et al. 2022, Adamczyk et al. 2025, Reķēna et al. 2025, Khuntong et al. 2025
*Cutaneotrichosporon oleaginosus*	Microbial oil	Efficient fatty acid production from various carbon sources	Duman-Özdamar et al. 2025
*Ogataea polymorpha*	Free fatty acids; ethanol	Thermotolerant; limited by-product accumulation; co-ferments pentoses and hexoses	Ryabova et al. 2003, Ni et al. 2024
*Kluyveromyces marxianus*	β-galactosidase, β-glucosidase, inulinase; single-cell protein; lactic acid, inositol; carotenoids; GABA; aroma compounds	Thermotolerant; rapid growth; multi-substrate assimilation	Fonseca et al. 2008, Karim et al. 2020, Baptista and Domingues 2022, Perpetuini et al. 2020, Yang et al. 2022, Lan et al. 2025
*Komagataella phaffii*	Malic acid, erythritol; antibody fragments, myo-inositol, silk fibroin nanoparticles; recombinant proteins (industrial enzymes, biopharmaceuticals and vaccines)	Efficient for C1 feedstocks; versatile high-cell-density protein expression platform	Guo et al. 2021, Wang et al. 2025a, Gómez-Ramírez et al. 2023, Zhang et al. 2022, Krishna et al. 2025, Tian et al. 2024, Unver Tian et al. 2024

## Future perspectives

**Figure 3:**
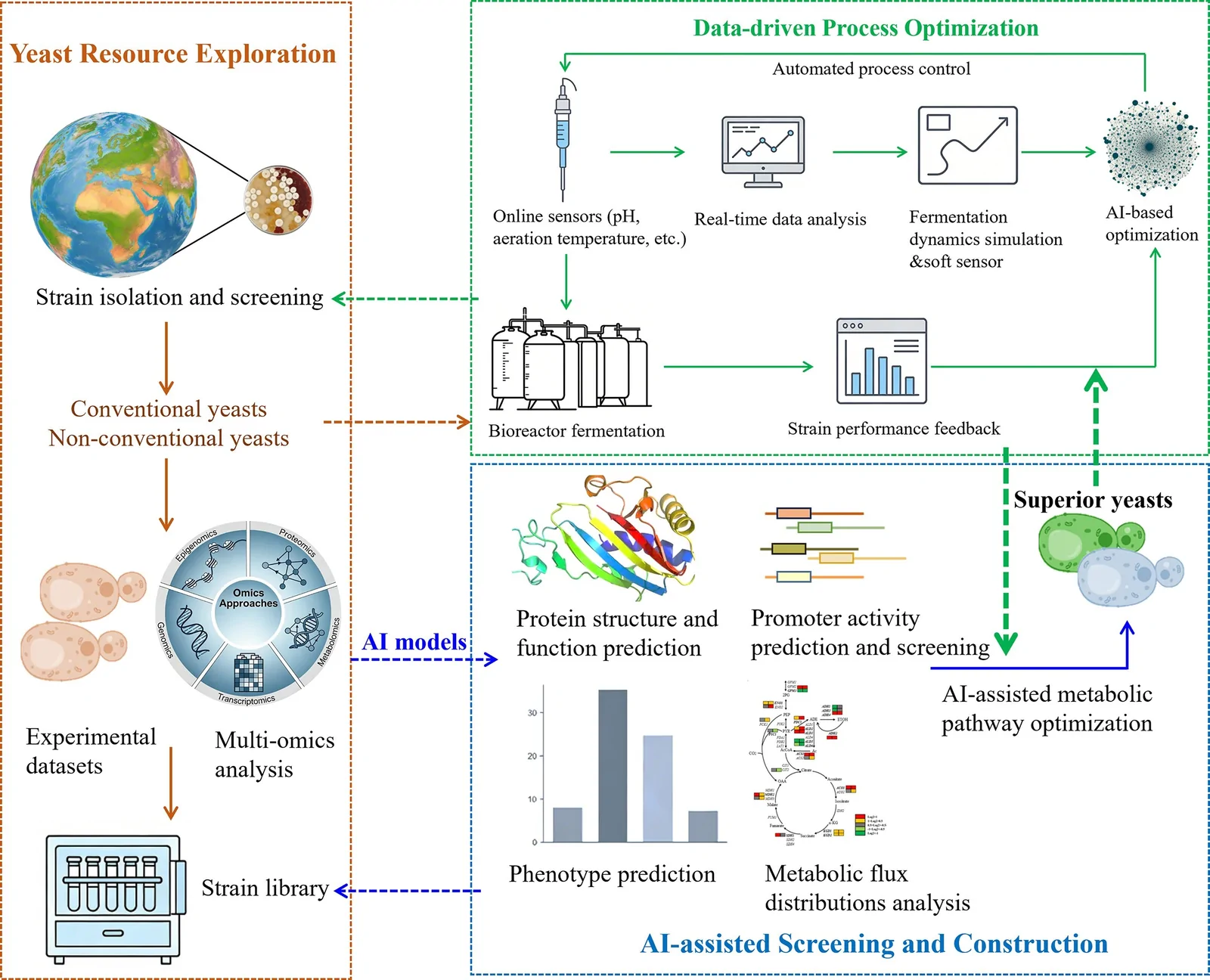
Schematic of the integrated workflow for yeast biodiversity-driven cell factory development. Novel yeast strains from diverse habitats are profiled by multi-omics to build strain libraries. Multiple AI tools enable prediction of protein function, promoter activity, metabolic flux, and strain phenotypes for rational construction of superior yeast strains. Engineered strains are tested in bioreactors under data-driven fermentation control, and fermentation performance data provides iterative feedback to optimize strain libraries and AI prediction models.

Despite the remarkable biodiversity of yeasts and recent progress in engineering strategies, several key challenges need to be overcome. These include the difficulty of genetic manipulation of most non-conventional yeasts and insufficient knowledge of their metabolic regulation. Product toxicity and a lack of large-scale fermentation experience are additional major constraints. Addressing these bottlenecks will require coordinated efforts in resource development, AI-assisted engineering, and process optimization, as outlined below (Fig. 3).

### Expanding non-conventional yeast resources and establishing strain libraries

It is essential to develop more non-conventional yeast resources and establish yeast resource libraries. Besides *S. cerevisiae*, many non-conventional yeast strains require further exploration, particularly those isolated from extreme environments. Crucially, physiological and metabolic diversity exists even at the strain level within the same species (Cheng et al. 2018). Therefore, the focus should be on building strain–level culture collections that encompass intraspecific variation, rather than merely depositing species–type strains. Establishing standardized culture collections encompassing strains from diverse habitats such as deep-sea ecosystems, and tropical zones will broaden application potential. Non-conventional yeasts demonstrate significant advantages in multi-substrate utilization, fermentation stress tolerance, and bioactive substances production. However, their genetic engineering shows greater challenges compared to *S. cerevisiae*, limiting their broader application. In recent years, advances in genome sequencing and genetic tool development enhance the feasibility of modifying these yeasts (Ganesan et al. 2019, Zha et al. 2023). As outlined in Fig. 3, the integration of genomics, transcriptomics, and metabolomics will deepen our understanding of functional genetic sites, unique metabolic pathways, and regulatory mechanisms in yeasts. Future efforts to decipher the integrated stress responses of extremophilic yeasts will accelerate the discovery of novel tolerance mechanisms and industrial hosts (Padilla-Garfias and Peña 2026). These insights will provide more targets and global regulatory strategies for the manipulation of not only non-conventional strains but also *S. cerevisiae*, facilitating the rational design of superior yeast cell factories.

### AI-assisted engineering of yeast cell factories

Artificial intelligence (AI) is expected to play an increasingly important role in yeast strain screening and engineering (Fig. 3). In data mining, AI extracts biological insights from large-scale omics and experimental datasets. For example, AI models predict ethanol yield under stress using cell morphology data (Itto-Nakama et al. 2021, Itto-Nakama et al. 2023). Biofoundries accelerate this via automated Design-Build-Test-Learn workflows (Martinez and Speight 2026). Combining protein language models like ESM-C, with structure prediction tools such as AlphaFold3 enables accurate prediction of protein properties such as solubility (Song et al. 2026).

In model prediction, AI enables predictive modeling of molecular and phenotypic outcomes. Deep learning models like Word2Vec combined with ResNet predict promoter activity in *S. cerevisiae* (Fu and Liu 2026). Hybrid approaches such as KineFlux combine machine learning with enzyme-constrained metabolic models to predict flux distributions from proteomic data (Soleymani et al. 2026). Machine learning models derived from large promoter libraries up to 675 000 constitutive and 327 000 inducible variants support rapid design of efficient promoters (Kotopka and Smolke 2020). Another study analyzed 100 million synthetic promoters to model transcription factor specificity and chromatin interactions (De Boer et al. 2020). In design and construction, generative AI and machine learning frameworks are applied to pathway optimization. A machine learning framework capturing interactions among metabolic modules is beneficial for iterative gene integration in *Y. lipolytica*, increasing β-carotene production seven-fold (Tu et al. 2026). AI-assisted biosensing contributes real-time fermentation monitoring (AlMarzooqi et al. 2025), and AI-driven segmentation automates morphological analysis for single-cell studies (Zhang et al. 2026). To provide a concise overview of the AI-assisted tools discussed above, we have summarized representative examples in Table 2.

**Table 2:** Selected AI-assisted tools and their applications in yeast engineering.

AI tool/framework	Application	References
Word2Vec-ResNet	Promoter activity prediction	Fu and Liu 2026
Generative ML/promoter libraries	Promoter design	Kotopka and Smolke 2020, De Boer et al. 2020
ESM-C + AlphaFold3	Protein structure and function prediction	Song et al. 2026
KineFlux (ML + enzyme-constrained GEM)	Metabolic flux prediction	Soleymani et al. 2026
Machine learning framework	Pathway optimization	Tu et al. 2026
AI-based morphology analysis	Fermentation performance prediction	Itto-Nakama et al. 2021, Itto-Nakama et al. 2023
AI-assisted biosensing	Real-time fermentation monitoring	AlMarzooqi et al. 2025

Together, these AI-driven approaches are conducive to the faster development of robust cell factories. The convergence of automation, machine learning, and synthetic biology is critical for construction of self-driving labs that transition from Design-Build-Test-Learn to Design-Build-Deploy workflows (Martinez and Speight 2026). Leveraging yeast biodiversity with AI will expand chassis and toolkits for next-generation metabolic engineering (Watcharawipas et al. 2025). Nevertheless, current AI applications in yeast engineering are constrained by the requirement for large, high-quality datasets, limited cross-species transferability, and limited mechanistic interpretability.

### Process engineering and optimization

Process design and fermentation optimization are critical to further improve the production capacity of engineered yeast strains. Even superior strains rely on process optimization to overcome scale-up limitations and system instability (Stadkowiski et al. 2026). Fermentation performance is influenced by multiple interacting parameters, including medium composition, temperature, pH, aeration, and feeding strategies. Accordingly, machine learning has emerged as a powerful tool for modeling complex system patterns based on experimental data. A standard workflow encompasses strategic experimental design to map the response landscape, followed by model training to simulate fermentation dynamics and identify optimal conditions (Wang et al. 2025b). In addition to basic optimization, advanced strategies such as automated process control, hybrid modeling, and soft sensor development have further broadened the applicability of machine learning in complex fermentation systems (Wang et al. 2025b). Besides fermentation optimization, downstream processing and process integration are key determinants of overall economic feasibility. For platform chemicals such as succinic acid, advances in separation technologies and integrated biorefinery design are necessary to reduce production costs and enhance sustainability (Bai et al. 2026). Therefore, integrating AI-assisted strain engineering with data-driven process optimization and efficient downstream recovery will ultimately facilitate the scale-up of lab innovations to economically sustainable biomanufacturing.

## Conclusion

With enhanced understanding of yeast biology and an expanded capacity for rational design, yeast platforms will increasingly serve as sustainable and versatile production systems for the bioeconomy. The integration of expanded natural diversity, advanced genetic tools, AI-assisted engineering, and data-driven process optimization will accelerate the development of yeast cell factories with desired diversity capable of converting low-cost and renewable feedstocks into a wide range of high-value products.
